# Polygenetic risk scores and phenotypic constellations of obsessive–compulsive disorder in clozapine-treated schizophrenia

**DOI:** 10.1007/s00406-023-01593-y

**Published:** 2023-04-05

**Authors:** Carla Lou Morgenroth, Philipp Kleymann, Stephan Ripke, Swapnil Awasthi, Elias Wagner, Tatiana Oviedo-Salcedo, Cynthia Okhuijsen-Pfeifer, Jurjen J. Luykx, Marte Z. van der Horst, Alkomiet Hasan, Felix Bermpohl, Stefan Gutwinski, Stefanie Schreiter

**Affiliations:** 1 Department of Psychiatry and Neurosciences, Charité - Universitätsmedizin Berlin, Corporate Member of Freie Universität Berlin and Humboldt-Universität Zu Berlin, Berlin, Germany; 2 Department of Psychiatry and Psychotherapy, University Hospital-LMU Munich, Munich, Germany; 3 Department of Psychiatry, University Medical Center Utrecht, Utrecht University, Utrecht, The Netherlands; 4 Brain Center Rudolf Magnus, Department of Translational Neuroscience, University Medical Center Utrecht, Utrecht, The Netherlands; 5 GGNet Mental Health, Warnsveld, The Netherlands; 6 Department of Psychiatry, Psychotherapy and Psychosomatics, Medical Faculty, University of Augsburg, Bezirkskrankenhaus Augsburg, Augsburg, Germany; 7 Department of Psychiatry, St. Hedwig-Krankenhaus, Charité-Universitätsmedizin, Berlin, Germany

**Keywords:** Schizophrenia, OCD, Clozapine, PRS

## Abstract

Obsessive–compulsive symptoms (OCS) are frequently observed in individuals with schizophrenia (SCZ) treated with clozapine (CLZ). This study aimed to analyze prevalence of OCS and obsessive–compulsive disorder (OCD) in this subgroup and find possible correlations with different phenotypes. Additionally, this is the first study to examine polygenetic risk scores (PRS) in individuals with SCZ and OCS. A multicenter cohort of 91 individuals with SCZ who were treated with CLZ was recruited and clinically and genetically assessed. Symptom severity was examined using the Positive and Negative Symptom Scale (PANSS), Clinical Global Impression Scale (CGI), the Calgary Depression Scale for Schizophrenia (CDSS), Global Assessment of Functioning Scale (GAF) and Yale–Brown Obsessive–Compulsive Scale (Y-BOCS). Participants were divided into subgroups based on phenotypic OCS or OCD using Y-BOCS scores. Genomic-wide data were generated, and PRS analyses were performed to evaluate the association between either phenotypic OCD or OCS severity and genotype-predicted predisposition for OCD, SCZ, cross-disorder, and CLZ/norclozapine (NorCLZ) ratio, CLZ metabolism and NorCLZ metabolism. OCS and OCD were frequent comorbidities in our sample of CLZ-treated SCZ individuals, with a prevalence of 39.6% and 27.5%, respectively. Furthermore, the Y-BOCS total score correlated positively with the duration of CLZ treatment in years (*r* = 0.28; *p* = 0.008) and the PANSS general psychopathology subscale score (*r* = 0.23; *p* = 0.028). A significant correlation was found between OCD occurrence and PRS for CLZ metabolism. We found no correlation between OCS severity and PRS for CLZ metabolism. We found no correlation for either OCD or OCS and PRS for OCD, cross-disorder, SCZ, CLZ/NorCLZ ratio or NorCLZ metabolism. Our study was able to replicate previous findings on clinical characteristics of CLZ-treated SCZ individuals. OCS is a frequent comorbidity in this cohort and is correlated with CLZ treatment duration in years and PANSS general psychopathology subscale score. We found a correlation between OCD and PRS for CLZ metabolism, which should be interpreted as incidental for now. Future research is necessary to replicate significant findings and to assess possible genetic predisposition of CLZ-treated individuals with SCZ to OCS/OCD. Limitations attributed to the small sample size or the inclusion of subjects on co-medication must be considered. If the association between OCD and PRS for CLZ metabolism can be replicated, it should be further evaluated if CYP1A2 alteration, respectively lower CLZ plasma level, is relevant for OCD development.

**Supplementary Information:**

The online version contains supplementary material available at 10.1007/s00406-023-01593-y.

## Introduction

Schizophrenia (SCZ) is a disorder with a multifaceted etiology that involves genetic and environmental factors, as well as their interactions [1–3]. Individuals with SCZ have a considerably higher risk of developing obsessive–compulsive disorder (OCD) or subclinical obsessive–compulsive symptoms (OCS). The prevalence of OCS among individuals with SCZ is estimated to be 25%, and about 12% of individuals with SCZ suffer from OCD, which is about ten times higher than that in the general population [4–7]. The prognosis of individuals with SCZ and OCS is worse than that of individuals without the comorbidity, e.g., due to lower social functioning or higher global, positive and negative symptoms [4, 8–10]. This co-occurrence has been particularly observed in individuals with SCZ who are treated with second-generation antipsychotics (SGA), especially clozapine (CLZ) [11]. Studies investigating the co-occurrence of OCS in individuals with SCZ treated with CLZ reveal prevalence rates up to 89% [1]. Data on the prevalence of OCD in individuals with SCZ under CLZ varies between 20% [12] and 47% [13]. Park et al. describe a de novo onset of OCD under CLZ in individuals with SCZ of 4.5% [14]. Thus, a causal correlation between comorbid OCD or OCS induction or exacerbation and the treatment of SCZ with SGA, especially with CLZ, is strongly hypothesized [15].

Several explanations for this association have been considered. One assumption is that SCZ patients with OCD represent a severely affected biological subtype and thus have a higher frequency of CLZ treatment [16]. Other explanations often regard the antiserotonergic and antidopaminergic properties of SGAs [17], which could exacerbate OCS by worsening or causing a serotonergic dysregulation [1]. The interference with glutamatergic neurotransmission should also be taken into account [8, 18]. Theories about the pathomechanisms of OCD development suggest that a serotonin dysregulation seems to be relevant because the treatment with selective serotonin reuptake inhibitor (SSRIs) medication leads to the improvement of OCS [1, 8, 19, 20]. Furthermore, glutamate, an excitatory neurotransmitter, has been identified to be increased in certain circuits of the brain, particularly in the cortico-striatal–thalamic circuits in individuals with OCD [21–23]. Research has shown that the inhibition of glutamate receptors can lead to a reduction of OCD symptoms [24]. CLZ is an antagonist of the serotonin receptor 5-hydroxytryptamine receptor 2A (5-HT2A) and dopamine receptors, with lower affinity to dopamine D_2_ receptors compared to first-generation antipsychotics [25]. Norclozapine (NorCLZ), the active metabolite of CLZ, acts on 5-HT2c receptors, which is associated with the appearance of side effects, modulates muscarinic 1 receptors, and could be an agonist on D_2_ and D_3_ receptors [25, 26].

Recent research suggests that the induction or exacerbation of OCS in (SGA-treated) individuals with SCZ may be explained by genetic risk constellations that predispose these individuals to OCD or OCS [1]. Specific genetic polymorphisms may make an individual with SCZ more sensitive to the pharmacological mechanisms that are thought to trigger or enhance the pathogenesis of OCS/OCD [1].

Thus far, four molecular genetic studies have been conducted to investigate the relationship between polymorphisms in targeted genes known to be related to OCD and the risk of developing OCS in SGA-treated SCZ cohorts. These studies have revealed that single nucleotide polymorphisms (SNPs) in SLC1A1, DLGAP3, and GRIN2B are associated with an increased risk of OCS in SGA-treated SCZ [18, 21, 27, 28]. However, replication studies are still lacking.

To date, only a few candidate genes and SNPs within these genes have been investigated to understand the genetic background of SGA-induced OCS in individuals with SCZ, despite that multiple genes and gene–gene interactions may be responsible for the development of SGA-induced OCS [27]. Complex illnesses are thought to arise from polygenetic pathways, meaning that they are not caused by a single gene, but by interactions between multiple risk loci. Recently, the analysis of polygenetic risk scores (PRS) has become more important in the understanding of the genetic etiology of psychiatric and other polygenic disorder. PRS are calculated as the weighted sums of all known risk variants for a particular disorder. In the context of genome-wide association studies (GWAS), all the genome-wide risk variants (SNPs) and their corresponding weights (effect sizes/odds ratio) from the largest available meta-analysis are used (as discovery data) to generate PRS profiles in a set of individuals with their SNPs genotyped (called target data). PRS profiles can explain the relative genetic susceptibility to a disease in the target cohort and can be used for risk prediction, screening and prevention [29].

To the best of our knowledge, no study has compared PRS between individuals with SCZ with and without OCS/OCD. In this proof-of-concept study, we performed PRS analyses to investigate the association between phenotypic occurrence of OCD or severity of OCS in individuals with SCZ treated with CLZ and genotype-predicted predisposition for the following traits: OCD, cross-disorder, SCZ and CLZ/NorCLZ ratio and metabolism. A positive correlation between these traits and our phenotype could help us understand the underlying pathology of OCD.

As several factors, such as genetic variations, tobacco, caffeine, and co-medication [26, 30], can individually impact CLZ and NorCLZ metabolism, plasma levels of CLZ and NorCLZ can vary, which has been associated with adverse reactions, such as OCS [31, 32]. To evaluate the impact of genetic alterations in metabolism on OCS/OCD, we used PRS analysis. CLZ is primarily metabolized in the liver by cytochrome P450 enzymes, including CYP1A2, CY2C19, CY3A4, and CYP2D6 [30, 33]. Pathways of metabolization include CLZ glucuronidation by UDP-glucuronosyltransferase (UGT), CLZ oxidation to clozapine-*N*-oxide by CYP3A4, and CLZ demethylation to its active metabolite NorCLZ by CYP1A2. The demethylation to NorCLZ by CYP1A2 is considered to be the predominant pathway and NorCLZ makes up most of the circulating CLZ concentration [30, 33]. Research has shown that NorCLZ alone has no antipsychotic effect, but is primarily responsible for adverse reactions to the drug [26, 33]. Since CLZ metabolism can vary individually due to inhibition (e.g., by fluvoxamine) or induction (e.g., by tobacco or caffeine) of CYP1A2, the monitoring of CLZ plasma concentration can be helpful to ensure that the therapeutic range (0.35–0.60 mg/L) is reached [26, 30]. NorCLZ plasmatic levels monitoring currently has no significance in clinical practice, but some studies suggest that it could be done to predict and avoid adverse effects [33, 34]. Genetic alterations that have been identified to impact CLZ metabolism are the CYP1A1/CYP1A2 SNP rs2472297, associated with lower CLZ plasma concentration, while the SNPs rs61750900 and rs2011425 in UGT genes have been linked to lower NorCLZ plasma levels [30]. An increase CLZ-to-NorCLZ ratio has been associated with the SNPs rs10023464 and rs7668556 in UGT genes and SNP rs12767583 in CYP2C19 [30].

Identifying the genetic predisposition of individuals with SCZ to OCS could help treating psychiatrists become more attentive to upcoming symptoms of OCD, enabling them to recommend targeted therapies or adapt the dosage for affected patients in the context of growing interest in precision medicine. The identification of genetic constellations associated with the development of OCS in individuals with SCZ treated with CLZ would be an important step towards personalized medicine. An individual treatment approach that implements more efficient therapies and minimizes risks is essential.

## Materials and methods

### Recruitment and study population

The study population was recruited as a part of the larger multicenter Clozapine International Consortium (CLOZIN), which is led by the University Medical Center Utrecht. This study aimed to investigate the underlying genetic architecture of treatment-resistant SCZ and identify clinical and genetic predictors of CLZ effectiveness and the occurrence of side effects [35]. The subsample was recruited from the Department of Psychiatry and Neurosciences at the Charité – Universitätsklinikum Berlin and at the Department of Psychiatry and Psychotherapy at the University Hospital of Munich (LMU Munich), in both inpatient and outpatient clinical settings from May 2017 to March 2020. The local ethics committees in Munich (Reference number 458-16) and Utrecht (Reference number 15-306) approved the study. The inclusion criteria were: (1) a diagnosis with schizophrenia, schizophreniform disorder, schizoaffective disorder, or psychosis not otherwise specified (NOS); (2) treatment with CLZ; (3) age above 18 years; (4) proficiency in the German language; (5) ability to provide informed consent. Exclusion criteria were: (1) admission to a psychiatric unit involuntarily in the context of an ‘inbewaringstelling’ (IBS), a particular compulsory treatment included in Dutch law, and (2) a history of Parkinson’s disease. A total number of 125 participants were recruited from the study centers in Berlin (*n* = 70) and Munich (*n* = 55), of whom 102 were eligible for the analyses of possible correlations of OCD and OCS severity and PRS. Twenty-three participants were excluded due to incomplete data.

### Clinical assessment and instruments

After obtaining informed consent, the participants underwent a structured interview and a blood draw, preferably combined with the monthly blood cell count check. Trained study raters conducted the interviews. The interviews included information on sociodemographic and clinical aspects, such as illness and medication history, substance use, and descendance from North-West Europe, which was a helpful criterion for genetic analyses. We used standardized instruments to assess symptom severity, including the Positive and Negative Symptom Scale (PANSS) [36], Yale–Brown Obsessive–Compulsive Scale (Y-BOCS) [37], Clinical Global Impression scale (CGI) [38], The Calgary Depression Scale for Schizophrenia (CDSS) [39], and overall functioning using the Global Assessment of Functioning Scale (GAF-Scale) [40]. The information obtained from the interviews was verified or supplemented by investigating the electronic patient files. To find possible correlations, although the criteria for OCD were not fully met, we also analyzed OCS severity [4]. Cutoff values for OCS/OCD were based on Y-BOCS scores. A cutoff score of 8 was defined for OCS (Y-BOCS ≥ 8) and a cutoff score of 13 for OCD (Y-BOCS ≥ 13), based on previous studies [28, 41].

### Statistical analyses of phenotypic data

We conducted descriptive analyses using SPSS (Version 26) [42]. To address the potential bias introduced by different study centers, we performed bivariate group testing between study centers. We also conducted bivariate group testing between participants with and without OCS and with and without OCD to identify differences in medication dosage or history, as well as illness history or symptom severity. We used *t* tests for normally distributed metric data, Mann–Whitney *U* tests for not normally distributed metric data, and Chi-square tests for nominal data. In addition, we evaluated the following variables for correlations using Pearson tests: Y-BOCS total score, PANSS total score, PANSS positive score, PANSS negative score, PANSS general score, GAF, CGI, CDSS, duration of CLZ treatment and prescribed dosage of CLZ. We chose Pearson tests instead of Spearman tests because our sample size was larger than 30 and we had no statistical outliers.

### Genetic analyses

#### Genotyping

We genotyped all blood samples in a single batch in Rotterdam using the Illumina Infinium^®^ Global Screening Array, version 3 (Illumina, San Diego, CA, USA). We conducted quality control (QC) with the genome analysis toolset PLINK v1.90b3z 64-bit (22 Nov 2015; https://www.cog-genomics.org/plink2version) and pre-imputation on the Michigan Imputation Server (https://imputationserver.sph.umich.edu), followed by post-imputation removal of all SNPs with a minor allele frequency (MAF) < 0.05 or an imputation score (*R*^2^) < 0.3 [35].

#### Quality control and imputation

We performed the technical and genomic QC and imputation using the GWAS pipeline RICOPILI [43]. The data of the final cohort of 102 participants was quality controlled in assent with the standards of the Psychiatric Genomics Consortium (PGC). To pass the QC, subjects and SNPs had to meet the following criteria: SNP missingness < 0.05 (before sample removal), subject missingness < 0.02, autosomal heterozygosity deviation (|Fhet|< 0.2), SNP missingness < 0.02 (after sample removal), difference in SNP missingness between cases and controls < 0.02, and SNP Hardy–Weinberg equilibrium (*p* > 10^−6^ in controls or *p* > 10^−10^ in cases). Population outliers were excluded by selecting a threshold from 2D plots of principal component 1 and 2 from a principal component analysis (PCA). PLINK v1.9 was used to test relatedness among the subject. We used 90,687 autosomal SNPs left after linkage disequilibrium pruning (*r*^2^ > 0.02) and frequency filtering of (MAF > 0.05) for testing. The pairs of subjects with PIHAT > 0.2 were identified and we excluded one member of each pair removed at random, preferably retaining cases. In summary, QC depicted/excluded three overlapping subjects, one subject with an ID call rate below 0.980, and seven subjects as population outliers (i.e., non-European ancestry with PCA1 > 0.1). In total, 11 individuals did not pass QC, resulting in a total sample of 91 participants (59 recruited in Berlin, 32 recruited in Munich).

The genotype imputation was conducted using the pre-phasing/imputation stepwise approach implemented in EAGLE/MINIMAC3 (with variable chunk size of 132 genomic chunks and default parameters) on 91 subjects (controls (no OCD) = 66, cases (with OCD) = 25). The imputation reference set consisted of 54,330 phased haplotypes with 36,678,882 variants from the publicly available HRC reference (https://ega-archive.org/datasets/EGAD00001002729).

#### Polygenetic risk scoring

PRS was performed for each subject using four central meta-analyses as training data [30, 44–46]. The training data included GWAS summary statistics for OCD, SCZ, cross-disorder and CLZ/NorCLZ ratio, CLZ metabolism and NorCLZ metabolism [30, 44–46]. The training data was LD pruned and “clumped” by discarding variants within 500 kb of, and in *r*^2^ ≥ 0.1 with another (more significant) marker. These LD-independent SNPs were used as weights to calculate the PRS of our targeted data (collected data), using various *p* value thresholds (pd): 5 × 10^–8^, 1 × 10^–6^, 1 × 10^–4^, 0.001, 0.01, 0.05, 0.1, 0.2, 0.5, and 1.0. Logistic regression of each variant was multiplied by the imputation probability for the risk allele in each subject. The resulting values were summed over each subject to obtain a whole-genome PRS for further analysis.

#### Linear and logistic regression analyses of OCD and PRS

A logistic regression and linear regression analyses were conducted to find possible correlations between PRS (for OCD, SCZ, CLZ/NorCLZ metabolism/ratio, cross-disorder) and clinically diagnosed OCD or OCS severity. Logistic regression was used to analyze the correlations between the occurrence of OCD and PRS, while linear regression searched for correlations between OCS severity and PRS. Regression models were adjusted for population stratification (using the principal components (PCs) 1–4 as covariates). The explained variance for the logistic regression analyses was estimated with Nagelkerke’s *R*^2^ (between predicted PRS and predicted and observed outcomes) by comparing scores generated from a full model (containing covariates and PRS) and a reduced model (covariates only). The beta coefficients and adjusted *R*^2^ for the multiple linear regression analyses were estimated using the RStudio programming software.

### Manuscript

We used the Large Language Model ChatGPT and the language assessment tool American Journal Experts (AJE) for grammar checking and English language revision of this paper.

## Results

### Sociodemographic and clinical characteristics of the study sample

The study sample consisted of 91 individuals, with a mean age of 42.8 years (SD = 10.6). Of these, 63.2% were male and 57.0% had a family history of psychiatric disorder. The majority (84.6%) were diagnosed with SCZ, while 14.3% with schizoaffective disorder and 1.1% with schizophreniform disorder. The mean illness duration was 17.9 years (SD = 10.3 years) and the mean daily dosage of CLZ was 244.03 mg (SD = 143.74) for a mean duration of 9.8 years (SD = 8.9). A total of 61.5% of the participants had fully descended from grandparents from North-West Europe. Additionally, 51.6% received co-medication with other SGAs in addition to CLZ, of which seven received aripiprazole (ARIP) (14.9%). Notably, all patients receiving ARIP were in the No-OCS/No-OCD group (STable 1).

Significant group differences between study centers were detected in the daily number of cigarettes, PANSS total score, as well as PANSS positive and general items score, and OCS severity. However, there was no group difference regarding the rates of OCD (STable 1).

### OCS severity and OCD in patients with SCZ

Of the 91 participants, 36 (39.6%) showed relevant OCS and 25 (27.5%) fulfilled criteria for OCD based on the predefined cutoff values [28, 41]. There was a significant group difference between OCS and non-OCS participants regarding the duration of CLZ treatment in years (OCS = 12.3 years, non-OCS = 8.0 years; *Z*(733.50) = − 1.97; *p* = 0.049) (Table 1). Participants with OCD were significantly older (OCD = 46.0 years, non-OCD = 41.5 years; *Z*(594.50) = − 2.05; *p* = 0.040), more likely to have co-medication with benzodiazepines/z-substances (OCD = 28.0%, non-OCD = 10.6%; × 2(1) = 4.21; *p* = 0.040), had a significantly higher PANSS general psychopathology subscale score (OCD = 35.4, non-OCD = 30.7; *T*(189) = − 2.34; *p* = 0.021), and had a significantly higher rate of grandparents from North-Western Europe (Z(595.50) = − 2.36; *p* = 0.018) (see Table 2). A significant positive correlation was observed between Y-BOCS total score and the duration of CLZ treatment in years (*r* = 0.28; *p* = 0.008).

**Table 1:** Comparison of sociodemographic and clinical characteristics of participants with and without obsessive–compulsive symptoms

Variables	Entire sample *N* (SD/%)	OCS (Y-BOCS ≥ 8)	No OCS (Y-BOCS < 8)	Group statistics
	*N* = 91	*N* = 36	*N* = 55	
Age in years	42.77 (± 10.57)	44.22 (± 11.07)	41.82 (± 10.22)	*Z*(839.50) = − 1.22; *p* = 0.221
Number of male participants	79 (63.2%)	25 (69.4%)	35 (63.6%)	*x*^2^(1) = 0.33; *p* = 0.568
Highest obtained degree				*x*^2^(1) = 0.01; *p* = 0.946
High school and/or above	35 (38.5%)	14 (38.9%)	21 (38.2%)	
Less than high school	56 (61.5%)	22 (61.1%)	34 (61.8%)	
Form of psychotic illness				*x*^2^(2) = 1.55; *p* = 0.462
Schizophrenia	77 (84.6%)	30 (83.3%)	47 (85.5%)	
Schizoaffective disorder	13 (14.3%)	5 (13.9%)	14.5 (14.5%)	
Schizophreniform disorder	1 (1.1%)	1 (2.8%)	0 (0.0%)	
Psychosis NOS	0 (0.0%)	0 (0.0%)	0 (0.0%)	
Duration of illness in years (*n* = 65)	17.88 (± 10.32)	21.05 (± 11.18) (*n* = 21)	16.36 (± 9.66) (*n* = 44)	*Z*(355.50) = − 1.50; *p* = 0.135
Duration of CLZ medication in years (*n* = 90)	9.77 (± 8.93)	12.32 (± 9.75)	8.07 (± 8.00) (n = 54)	***Z*(733.50) = **− **1.97; *p* = 0.049**
Prescribed daily dosage CLZ in mg (*n* = 90)	244.03 (± 143.74)	231.42 (± 140.95) (*n* = 35)	252.05 (± 146.21)	*T*(88) = 0.66; *p* = 0.510
Co-medication				
Number of participants with antidepressant medication^1^	24 (26.4%)	11 (30.6%)	13 (23.6%)	*x*^2^(1) = 0.54; *p* = 0.464
Number of participants with anticonvulsant medication/mood stabilizers^2^	22 (24.2%)	9 (25.0%)	13 (23.6%)	*x*^2^(1) = 0.02; *p* = 0.882
Number of participants with first-generation antipsychotic medication^3^	24 (26.4%)	6 (16.7%)	18 (32.7%)	*x*^2^(1) = 2.89; *p* = 0.089
Number of participants with second-generation antipsychotics^4^	47 (51.6%)	18 (50.0%)	29 (52.7%)	*x*^2^(1) = 0.07; *p* = 0.799
Number of participants with benzodiazepines and z-substances^5^	14 (15.4%)	7 (19.4%)	7 (12.7%)	*x*^2^(1) = 0.75; *p* = 0.385
Daily number of cigarettes	11.38 (± 14.35)	13.32 (± 17.29)	9.82 (± 11.97)	*Z*(914.50) = − 0.66; *p* = 0.511
Daily consumption of coffee in cups	3.24 (± 2.67)	3.33 (± 3.22)	3.18 (± 2.28)	*Z*(952.00) = − 0.31; *p* = 0.758
Number of participants with a family history for psychiatric disorder (*n* = 86)	49 (57.0%)	19 (55.9%) (*n* = 34)	30 (57.7%) (*n* = 52)	*x*^2^(1) = 0.03; *p* = 0.868
Number of grandparents from North-West Europe	2.75 (± 1.73)	3.11 (± 1.55)	2.51 (± 1.81)	*Z*(809.00) = − 1.70; *p* = 0.090
0 Grandparents from North-West Europe	23 (25.3%)	6 (16.7%)	17 (30.9%)	*x*^2^(4) = 5.83; *p* = 0.212
1 Grandparents from North-West Europe	1 (1.1%)	0 (0.0%)	1 (1.8%)	
2 Grandparents from North-West Europe	8 (8.8%)	4 (11.1%)	4 (7.3%)	
3 Grandparents from North-West Europe	3 (3.3%)	0 (0.0%)	3 (5.5%)	
4 Grandparents from North-West Europe	56 (61.5%)	26 (72.2%)	30 (54.5%)	
PANSS total score	62.49 (± 16.21)	63.83 (± 17.63)	61.62 (± 15.32)	*Z*(948.50) = − 0.34; *p* = 0.736
PANSS positive items	15.15 (± 5.12)	15.61 (± 5.69)	14.85 (± 4.74)	*Z*(931.50) = − 0.48; *p* = 0.634
PANSS negative items	15.43 (± 5.33)	14.61 (± 4.96)	15.96 (± 5.53)	*T*(89) = 1.19; *p* = 0.238
PANSS general items	32.00 (± 8.73)	33.58 (± 9.14)	30.96 (± 8.38)	*T*(89) = − 1.41; *p* = 0.163
CGI	4.36 (± 1.03)	4.33 (± 1.07)	4.38 (± 1.01)	*Z*(971.00) = − 0.16; *p* = 0.873
GAF (*n* = 90)	51.64 (± 13.41)	50.14 (± 13.52)	52.65 (± 13.42) (*n* = 54)	*T*(88) = 0.87; *p* = 0.389
CDSS	4.27(± 4.13)	4.42 (± 4.18)	4.18 (± 4.14)	*Z*(929.50) = − 0.50; *p* = 0.621
Inpatient setting (*n* = 59)	17 (28.8%)	6 (21.4%) (*n* = 28)	11 (35.5%) (*n* = 31)	*x*^2^(1) = 1.42; *p* = 0.234

Significant results are marked in bold

CDSS, Calgary Depression Scale for Schizophrenia; CGI, Clinical Global Impression; CLZ, clozapine; GAF, Global Assessment of Functioning Scale; OCD, obsessive–compulsive disorder; OCS, obsessive–compulsive symptoms; PANSS, Positive and negative Symptoms Scale; Psychosis NOS, psychosis not otherwise specified; Y-BOCS, Yale–Brown Obsessive–Compulsive Scale

^1^Milnacipran, escitalopram, venlafaxine, sertraline, doxepin, citalopram, fluvoxamine, paroxetine, duloxetine, amitriptyline

^2^Valproate, pregabalin, levetiracetam, lamotrigine, lithium

^3^Haloperidol, pipamperone, chlorprothixene, promethazine, melperon, perazin, levomepromazine, ziprasidone, loxapin, flupentixol

^4^Paliperidone, risperidone, amisulprid, aripiprazole, olanzapine, quetiapine

^5^Diazepam, lorazepam, zopiclone, clobazam

**Table 2:** Comparison of sociodemographic and clinical characteristics of participants with and without obsessive–compulsive disorder

Variables	Entire sample *N* (SD/%)	OCD (Y-BOCS ≥ 13)	No OCD (Y-BOCS < 13)	Group statistics
*N* = 91	*N* = 91	*N* = 25	*N* = 66	
Age in years	42.77 (± 10.57)	46.00 (± 11.39)	41.55 (± 10.06)	***Z*(594.50) = **− **2.05; *p* = 0.040**
Number of male participants	79 (63.2%)	18 (72.0%)	42 (63.6%)	*x*^2^(1) = 0.57; *p* = 0.452
Highest obtained degree				*x*^2^(1) = 0.03; *p* = 0.853
High school and/or above	35 (38.5%)	10 (40.0%)	25 (37.9%)	
Less than high school	56 (61.5%)	15 (60.0%)	41 (62.1%)	
Form of psychotic illness				*x*^2^(2) = 0.55; *p* = 0.759
Schizophrenia	77 (84.6%)	22 (88.0%)	55 (83.3%)	
Schizoaffective disorder	13 (14.3%)	3 (12.0%)	10 (15.2%)	
Schizophreniform disorder	1 (1.1%)	0 (0.0%)	1 (1.5%)	
Psychosis NOS	0 (0.0%)	0 (0.0%)	0 (0.0%)	
Duration of illness in years (*n* = 65)	17.88 (± 10.32)	21.36 (± 13.04) (*n* = 14)	16.92 (± 9.38) (*n* = 51)	*Z*(296.00) = − 0.97; *p* = 0.330
Duration of CLZ medication in years (*n* = 90)	9.77 (± 8.93)	12.94 (9.87)	8.55 (± 8.31) (*n* = 65)	*Z*(612.00) = − 1.81; *p* = 0.070
Prescribed daily dosage CLZ in mg (*n* = 90)	244.03 (± 143.74)	251.00 (± 150.43)	241.35 (± 142.20) (*n* = 65)	*T*(88) = − 0.28; *p* = 0.777
Co-medication				
Number of participants with antidepressant medication^1^	24 (26.4%)	9 (36.0%)	15 (22.7%)	*x*^2^(1) = 1.65; *p* = 0.200
Number of participants with anticonvulsant medication/mood stabilizers^2^	22 (24.2%)	5 (20.0%)	17 (25.8%)	*x*^2^(1) = 0.33; *p* = 0.567
Number of participants with first-generation antipsychotic medication^3^	24 (26.4%)	3 (12.0%)	21 (31.8%)	*x*^2^(1) = 3.67; *p* = 0.055
Number of participants with second-generation antipsychotics^4^	47 (51.6%)	13 (52.0%)	34 (51.5%)	*x*^2^(1) = 0.00; *p* = 0.967
Number of participants with benzodiazepines and z-substances^5^	14 (15.4%)	7 (28.0%)	7 (10.6%)	***x***^**2**^**(1) = 4.21; *p* = 0.040**
Daily number of cigarettes	11.38 (± 14.35)	14.56 (± 18.50)	10.18 (± 12.39)	*Z*(762.00) = − 0.60; *p* = 0.548
Daily consumption of coffee in cups	3.24 (± 2.67)	3.56 (± 3.34)	3.12 (± 2.39)	*Z*(803.00) = − 0.198; *p* = 0.843
Number of participants with a family history for psychiatric disorder (*n* = 86)	49 (57.0%)	12 (52.2%) (*n* = 23)	37 (58.7%) (*n* = 63)	*x*^2^(1) = 0.30; *p* = 0.587
Number of grandparents from North-West Europe	2.75 (± 1.73)	3.40 (± 1.30)	2.48 (± 1.83)	***Z*(595.50) = **− **2.36; *p* = 0.018**
0 grandparents from North-West Europe	23 (25.3%)	2 (8.0%)	21 (31.8%)	*x*^2^(4) = 7.90; *p* = 0.095
1 grandparents from North-West Europe	1 (1.1%)	0 (0.0%)	1 (1.5%)	
2 grandparents from North-West Europe	8 (8.8%)	3 (12.0%)	5 (7.6%)	
3 grandparents from North-West Europe	3 (3.3%)	0 (0.0%)	3 (4.5%)	
4 grandparents from North-West Europe	56 (61.5%)	4 (80.0%)	36 (54.5%)	
PANSS total score	62.49 (± 16.21)	67.48 (± 18.77)	60.61 (± 14.86)	*Z*(648.00) = − 1.58; *p* = 0.115
PANSS positive items	15.15 (± 5.12)	16.28 (± 5.98)	14.73 (± 4.74)	*Z*(718.50) = − 0.95; *p* = 0.342
PANSS negative items	15.43 (± 5.33)	15.76 (± 5.26)	15.30 (± 5.38)	*T*(89) = − 0.36; *p* = 0.717
PANSS general items	32.00 (± 8.73)	35.40 (± 9.89)	30.71 (± 7.96)	***T*(189) = **− **2.34; *p* = 0.021**
CGI	4.36 (± 1.03)	4.44 (± 1.04)	4.33 (± 1.03)	*Z*(784.50) = − 0.37; *p* = 0.708
GAF (*n* = 90)	51.64 (± 13.41)	47.68 (± 13.40)	53.17 (± 13.24) (*n* = 65)	*T*(88) = − 1.76; *p* = 0.083
CDSS	4.27(± 4.13)	4.88 (± 4.71)	4.05 (± 3.90)	*Z*(746.00) = − 0.71; *p* = 0.479
Inpatient setting (*n* = 59)	17 (28.8%)	6 (30.0%) (*n* = 20)	11 (28.2%) (*n* = 39)	*x*^2^(1) = 0.02; *p* = 0.885

Significant results are marked in bold

CDSS, Calgary Depression Scale for Schizophrenia; CGI, Clinical Global Impression; CLZ, clozapine; GAF, Global Assessment of Functioning Scale; OCD, obsessive–compulsive disorder; OCS, obsessive–compulsive symptoms; PANSS, Positive and Negative Symptoms Scale; Psychosis NOS, psychosis not otherwise specified; Y-BOCS, Yale–Brown Obsessive–Compulsive Scale

^1^Milnacipran, escitalopram, venlafaxine, sertraline, doxepin, citalopram, fluvoxamine, paroxetine, duloxetine, amitriptyline

^2^Valproate, pregabalin, levetiracetam, lamotrigine, lithium

^3^Haloperidol, pipamperone, chlorprothixene, promethazine, melperon, perazin, levomepromazine, ziprasidone, loxapin, flupentixol

^4^Paliperidone, risperidone, amisulprid, aripiprazole, olanzapine, quetiapine

^5^Diazepam, lorazepam, zopiclone, clobazam

### Genetic results

91 individuals and 90,687 LD clumped autosomal SNPs passed the QC and were included in the GWAS and PRS analyses. Results for the binary logistic regression (OCD vs. non-OCD) and the multiple linear regression (OCS severity based on Y-BOCS score) can be found in Tables 3 and 4. We evaluated the correlation at ten different *p* value thresholds. A nominally significant result was found for the logistic regression (OCD vs. non-OCD) of CLZ metabolism (*p* = 0.010) at pd = 0.001, which is listed in Table 3. When correcting for multiple testing using Bonferroni correction for only the CLZ metabolism phenotype (10 tests), the result remained significant, whereas it was no longer significant when correcting for all six phenotypes (60 tests). The new significance threshold when correcting only for the CLZ metabolism phenotype was 0.005. No other significant correlations between the PRS for the different phenotypes and OCD diagnosis (logistic regression) was detected. No correlation between OCS severity (multiple linear regression) and PRS for CLZ metabolism, CLZ/NorCLZ ratio, NorCLZ metabolism, cross-disorder or SCZ was found.

**Table 3:** Logistic regression of PRS and OCD diagnosis (adjusted for population stratification and corrected for PCs). Sources: [30, 44–46]

Phenotype/PRS	NBIN	pd	*N*	NKr2	*p*val	Ncase	Ncontrol	Coeff with cov
OCD	105,119	1.0	91	0.018	0.285	25	66	− 0.299
SCZ	98,963	1.0	91	0.004	0.635	25	66	0.122
CLZ metabolism	141,156	1.0	91	9.25E−05	0.940	25	66	− 0.019
	778	0.001	91	0.103	**0.010**	25	66	0.654
CLZ/NORCLZ ratio	141,225	1.0	91	0.012	0.387	25	66	0.231
Norclozapine metabolism	141,052	1.0	91	0.021	0.251	25	66	− 0.315
CDO	78,254	1.0	91	0.015	0.328	25	66	0.264

Significant results are marked in bold

CDO, cross-disorder; CLZ, clozapine; Coeff_with_cov, coefficient of regression (for direction of effect); *N*, number of individual tested; NBIN, number of SNPs used to generate PRS at this *p* value threshold; Ncase, number of cases in *N*; Ncontrols, number of controls in *N*; NKr2, the Nagelkerke *R*^2^; NORCLZ, norclozapine; OCD, obsessive–compulsive disorder; PCs, principal components; PD, *p* value threshold; PRS, polygenetic risk score; *P*val, significance of the correlation; SCZ schizophrenia

**Table 4:** Multiple linear regression of PRS and Y-BOCS total score (adjusted for population stratification and corrected for PCs). Sources: [30, 44–46]

Phenotype/PRS	NBIN	pd	N	Adjusted *R*^2^	*p*val	Coeff estimate	Std. error
OCD	105,119	1.0	91	− 0.040	0.903	5.734	5.517
SCZ	98,963	1.0	91	− 0.040	0.901	19.820	39.873
CLZ metabolism	141,156	1.0	91	− 0.037	0.876	7.441	0.989
CLZ/NORCLZ ratio	141,225	1.0	91	− 0.034	0.840	7.210	0.960
Norclozapine metabolism	141,052	1.0	91	− 0.039	0.900	7.152	1.053
CDO	78,254	1.0	91	− 0.025	0.726	31.121	20.755

CDO, cross-disorder; CLZ, clozapine; Coeff_with_cov, coefficient of regression (for direction of effect); *N*, number of individual tested; NBIN, number of SNPs used to generate PRS at this *p* value threshold; Ncase, number of cases in *N*; Ncontrols, number of controls in *N*; NKr2, the Nagelkerke *R*^2^; NORCLZ, norclozapine; OCD, obsessive–compulsive disorder; PCs, principal components; PD, *p* value threshold; PRS, polygenetic risk score; *P*val, significance of the correlation; SCZ schizophrenia

## Discussion

The present proof-of-concept study is the first to compare PRS in individuals with SCZ treated with CLZ with and without OCS or OCD. The study included 91 participants and analyzed 90,687 autosomal SNPs.

In terms of clinical findings, the study’s cohort of CLZ-treated participants had significantly higher rates of OCS and OCD compared to the general population. The prevalence of OCD was 27.5% among our cohort, which is higher than the lifetime prevalence of OCD of 1.3% [47] reported in the general population. This finding is consistent with previous studies of individuals with SCZ treated with CLZ, which have reported OCD prevalence rates ranging from 20 to 47% [1, 12, 13, 48]. Additionally, 39.6% of the participants in this study fulfilled criteria for OCS. It is important to note that OCD may not always be diagnosed consistently across different studies. While OCD is a clinical diagnosis that does not rely solely on the Y-BOCS score, there may be variations in diagnostic criteria and methodologies used in different studies.

We were able to replicate the previous finding of a significant correlation between the duration of CLZ treatment and OCS severity, which is in line with former studies [1, 14]. Schirmbeck et al. found a positive association between OCS severity and duration of CLZ treatment [48], and Scheltema Beduin et al. observed that OCS frequency increased when CLZ was taken for over 6 months compared to an intake of less than 6 months [49, 50]. Park et al. found that the mean time from CLZ initiation to the appearance of OCD was 1.9 years, with 34% of patients being diagnosed with OCD after 12 months of CLZ treatment, and 57% after 24 months [14]. In line with Lin et al., we found a significant correlation between OCS severity and the duration of CLZ treatment, but not with duration of illness [8, 31], supporting the theory that CLZ has an influence on OCS development in SCZ. However, Ertugrul et al. did not find an association between CLZ treatment duration and OCS development [51], although most studies, including the present one, show this correlation. While several studies have reported a correlation between CLZ dosage and OCS [1, 8, 48, 52], our study did not replicate these findings, consistent with the study by Ertugrul et al. [51]. This may be attributed to the impact of other factors on CLZ metabolism, such as smoking and caffeine, which can affect CLZ blood levels, causing them to deviate from the prescribed CLZ dosage [30, 53, 54].

Our study found a significant correlation between OCS severity and the PANSS general psychopathology subscale score, which is consistent with previous studies [15, 55, 56]. We detected no correlation of OCS severity and PANSS positive or negative symptom scale. Schirmbeck et al. reported an association between Y-BOCS score and PANSS general subscale score in individuals treated with CLZ or olanzapine [15]. However, the reason for this correlation is not yet fully understood [15], but it may be related to higher overall symptom severity, as well as affective and depressive symptoms observed in individuals with SCZ and comorbid OCS [1, 8, 15].

Our genetic analyses suggest a potential correlation between phenotypic of OCD and the PRS for CLZ metabolism. However, as the significant result only appeared at one *p* value threshold and did not survive correction for multiple testing for all 6 phenotypes, it is most likely a random finding.

SNPs from the central meta-analyses on CLZ and NorCLZ metabolism indicate that reduced plasma concentration of CLZ and NorCLZ is associated with altered enzyme activity [30]. The study found SNPs affecting CYP1A2 activity to have a relevant effect on CLZ metabolism. CYP1A2 has been shown to play a major role in the in vivo metabolism of CLZ by oxidating CLZ to NorCLZ (active metabolite) and clozapine-*N*-oxide (inactive metabolite) [57]. Okhuijsen-Pfeifer et al. recently found that genotype-predicted CYP1A2 activity is inversely associated with dose-adjusted CLZ levels, but not with symptom severity [35]. However, other studies do suggest an association between CYP1A2 enzyme activity and symptom severity [58]. The relationship between CLZ plasma levels and clinical response is not fully understood [54]. Our findings suggest that PRS of CYP1A2 and thus reduced plasma concentration of CLZ may be associated with OCD.

One possible explanation for this association could be that CLZ is more thoroughly metabolized to the active metabolite NorCLZ, which has been found to cause adverse reactions while having fewer antipsychotic effects [35, 59]. However, whether NorCLZ causes OCD as a side effect requires further investigation. Previous studies have shown a correlation between NorCLZ plasma levels and OCS, but these studies also found a correlation between CLZ plasma levels and OCS, which is not in line with our findings [31, 32]. Other studies have shown that co-administration of CLZ and the SSRI fluvoxamine can decrease adverse reaction to CLZ and enhance clinical response. This may be linked to fluvoxamine’s inhibition of CYP1A2, which increases CLZ and NorCLZ plasma levels. This augmentation is even measurable when CLZ-treated individuals smoke tobacco, which typically decreases serum concentrations by inducing CYP1A2 [26].

The PRS analysis did not reveal an association between the CLZ/NorCLZ ratio and OCS/OCD. The relevance of CLZ/NorCLZ ratio is unclear. In fact, a recent review by Schoretsanitis et al. concluded that CLZ/NorCLZ ratio is not correlated with clinical response to CLZ and is not a measure of CYP1A2 activity. Further, it is not significantly influenced by tobacco smoking [33]. Other studies have shown that an increased NorCLZ/CLZ ratio is associated with improved clinical outcomes [60].

We expected to find a positive correlation between the PRS for OCD and our OCD/OCS phenotype; however, none was found. Next to methodological limitations, it is also debatable if there are other genetic setups correlated to OCS/OCD than to SGA-induced OCS/OCD. Previous research has suggested that OCD in individuals with SCZ may not solely be induced by SGAs but may be attributed to the existence of a “schizo-obsessive” subtype of SCZ or a “schizotypic OCD” condition [17]. If these subtypes exist, then it is necessary to take into account the different genetic makeup of each category; however, we did not differentiate between the possible subtypes in our study.

It has been suggested that cross-disorder PRS and SCZ-PRS may be associated with lower SCZ symptom severity in CLZ-treated individuals with SCZ, possibly due to a better response to treatment [35]. Future research is needed to determine whether a better response to CLZ can also affect OCS development in individuals with SCZ.

### Limitations

To form a homogenous subgroup, our study only included individuals with a DSM-based SCZ diagnosis who were treated with CLZ, since previous PRS analysis suggests that these individuals have a more homogenous genetic set up [16]. However, this limits the number of participants, which is a significant limitation of our study. Additionally, there were some clinical differences between the study centers, including differences in tobacco smoking, which is important because tobacco smoking can induce the activity of CYP1A2 [53, 54]. Furthermore, co-medication with other SGAs or SSRIs may have influenced the phenotype. OCS can be regressive under treatment with SSRIs [1, 19, 20] or progressive under other SGAs than CLZ [12], except for ARIP, which may reduce OCS [61–64]. 26.4% of our entire sample was co-medicated with antidepressants, including SSRIs, which are known to reduce OCS and are indicated for the treatment of OCD [1, 8]. Furthermore, 51.6% of our sample was co-medicated with other SGAs than CLZ, of which some are also known to induce OCS. On the other hand, the SGA ARIP is also a potential treatment for OCS and SGA-induced OCS [61, 62], especially when combined with SSRIs [63, 64]. All patients on ARIP (7.7%) were in the No-OCD/OCS group. These individuals could carry genetic variations related to OCS/OCD that were not included in the OCS/OCD group. This may have affected our PRS analyses. Excluding patients on other antipsychotic or antidepressant medication can be considered in future studies to minimize bias, although this will reduce sample size even more, as well as generalizability of the results as this group often receives polypharmacy.

Lastly, our study design is cross-sectional and participants without OCS at the time of the interview may develop OCS in the future. This is in line with previous findings that suggest a positive correlation between OCS occurrence and treatment duration [8, 14, 48]. In the OCS/OCD groups, information about the presence of OCS before CLZ initiation is missing. It is possible that subjects already showed OCS before CLZ treatment. In this case, OCS could not be viewed as an adverse reaction to CLZ; yet, CLZ could still have enhanced preexisting OCS.

Additionally, we did not include a comparison group of individuals with SCZ who received other SGA treatment, despite evidence indicating a higher prevalence of OCD among those treated with olanzapine [49].

## Conclusion

Individuals with SCZ who receive CLZ treatment represent a homogenous subgroup of individuals with SCZ, since CLZ is usually only prescribed to subjects with treatment-resistant SCZ. In line with former research OCS and OCD were common in our sample and a significant correlation between OCS severity and CLZ treatment duration was detected, which supports the idea that CLZ can induce OCS. Also, we found a correlation between OCS severity and PANSS general psychopathology subscale score.

Finding a genetic explanation for the frequent comorbidity of OCD among individuals with SCZ treated with CLZ is a promising attempt to understand and possibly avoid this co-occurrence, which would be step towards personalized medicine. Our proof-of-concept study is the first to calculate PRS for 91 participants in this subgroup and evaluate correlations with OCD, SCZ, cross-disorder and CLZ/NorCLZ ratio and metabolism. We found a significant association between OCD and PRS for CLZ metabolism, which was most likely an incidental finding. No additional association were found in the PRS analyses, including no significant association between OCS severity and PRS for CLZ metabolism. If this association between OCD and PRS for CLZ metabolism, however, could be proven in the future, it could lead to the postulation that CYP1A2 alteration and thus lower CLZ plasma levels may influence OCD development. However, the current literature suggests a correlation between higher CLZ levels and OCS development and severity [31, 32], which is inconsistent with our results. Additionally, we did not find a correlation between SGA-induced OCS/OCD and the PRS for OCD, which could be due to methodological factors or differences in genetic backgrounds. The major limitations of our studies were the small sample size and co-medication with SSRIs and other SGAs.

Future studies with larger sample sizes and the possible exclusion of co-medication with SSRIs and SGAs need to be conducted to confirm and replicate our findings and achieve more powerful and representative results in a GWAS looking for genetic loci associated with OCD in SCZ treated with CLZ. Longitudinal studies with a long-term follow-up are also necessary to investigate when patients develop OCD and the influence of treatment duration.

### Supplementary Information

Below is the link to the electronic supplementary material.

Supplementary file1 (DOCX 61 KB)

## Data Availability

Raw data for all datasets are not publicly available to preserve individuals’ privacy under the European General Data Protection Regulation.
