# Supplementary material for: Polygenetic risk scores and phenotypic constellations of obsessive–compulsive disorder in clozapine-treated schizophrenia
Source: Eur Arch Psychiatry Clin Neurosci. 2023 Apr 5;274(1):181–93. doi: 10.1007/s00406-023-01593-y (PMC10786740; doi:10.1007/s00406-023-01593-y)
Supplement: Supplementary file 1 — Supplementary file1 (DOCX 61 KB) [file 406_2023_1593_MOESM1_ESM.docx]

**STable 1** – Sociodemographic and clinical characteristics of the entire sample and comparison of study centers

| **Variables** | **Study Center** | | **Group Statistics** |
| --- | --- | --- | --- |
|  | **Berlin** | **Munich** |  |
| **N=91** | N=59 | N=32 |  |
| Age in years | 42.27 (±10.77) | 43.69 (±10.29) | Z(896.00)=-0.40; p=0.690 |
| Number of male participants | 39 (66.1%) | 21 (65.6%) | x^2^(1)=0.002; p=0.963 |
| Highest obtained degree |  |  | x^2^(1)=2.78; p=0.096 |
| Highschool and/or above | 19 (32.2%) | 16 (50.0%) |  |
| Less than Highschool | 40 (67.8%) | 16 (50.0%) |  |
| Form of Psychotic Illness |  |  | x^2^(2)=0.70; p=0.704 |
| Schizophrenia | 49 (83.1%) | 28 (87.5%) |  |
| Schizoaffective disorder | 9 (15.3%) | 4 (12.5%) |  |
| Schizophreniform disorder | 1 (1.7%) | 0 (0.0%) |  |
| Psychosis NOS | 0 (0.0%) | 0 (0.0%) |  |
| Duration of Illness in years (n=65) | 17.91 (±10.41) (n=33) | 17.84 (±10.40) | Z(515.50)=-0.16; p=0.870 |
| Duration of CLZ medication in years (n=90) | 9.59 (±9.44) | 10.10 (±8.03) (n=31) | Z(847.00)=-0.57; p=0.566 |
| Prescribed daily Dosage CLZ in mg (n=90) | 259.91 (±142.04) (n=58) | 215.23 (±144.55) | T(88)=1.42; p=0.159 |
| Comedication |  |  |  |
| Number of participants with antidepressant medication ^[[1]](#footnote-1)^ | 16 (27.1%) | 8 (25.0%) | x^2^(1)=0.05; p=0.827 |
| Number of participants with anticonvulsant medication/ mood  stabilizers ^[[2]](#footnote-2)^ | 15 (25.4%) | 7 (21.9%) | x^2^(1)=0.14; p=0.706 |
| Number of participants with first generation antipsychotic  medication ^[[3]](#footnote-3)^ | 18 (30.5%) | 6 (18.8) | x^2^(1)=1.48; p=0.224 |
| Number of participants with second generation antipsychotics^[[4]](#footnote-4)^ | 30 (50.8%) | 17 (53.1%) | x^2^(1)=0.40; p=0.836 |
| Number of participants with benzodiazepines and z-  Substances ^[[5]](#footnote-5)^ | 7 (11.9%) | 7 (21.9%) | x^2^(1)=1.60; p=0.206 |
| Daily number of cigarettes | 14.92 (±15.29) | 4.88 (±9.65) | **Z(549.50)=-3.52; p<0.001** |
| Daily consumption of coffee in cups | 3.51 (±2.67) | 2.75 (±2.65) | Z(754.50)=-1.59; p=0.111 |
| Number of participants with a family history for psychiatric disorder (n=86) | 32 (58.2%) (n=55) | 17 (54.8%) (n=31) | x^2^(1)=0.09; p=0.764 |
| Number of Grandparents from North-West Europe | 2.86 (±1.68) | 2.53 (±1.83) | Z(854.50)=-0.86; p=0.390 |
| 0 Grandparents from North-West Europe | 13 (22.0%) | 10 (31.3%) | x^2^(4)=1.49; p=0.829 |
| 1 Grandparents from North-West Europe | 1 (1.7%) | 0 (0.0%) |  |
| 2 Grandparents from North-West Europe | 5 (8.5%) | 3 (9.4%) |  |
| 3 Grandparents from North-West Europe | 2 (3.4%) | 1 (3.1%) |  |
| 4 Grandparents from North-West Europe | 38 (64.4%) | 18 (56.3%) |  |
| PANSS Total score | 66.05 (±17.51) | 55.94 (±11.48) | **Z(625.50)=-2.65; p=0.008** |
| PANSS positive items | 16.24 (±5.58) | 13.16 (±3.39) | **Z(655.50)=-2.41; p=0.016** |
| PANSS negative items | 15.66 (±5.57) | 15.00 (±4.90) | T(89)=0.56; p=0.575 |
| PANSS general items | 34.29 (±9.07) | 27.78 (±6.27) | **T(83.65) = -24.02; p<0.001** |
| CGI | 4.44 (±1.13) | 4.22 (±0.79) | Z(842.50) = -0.88; p=0.380 |
| GAF (n=90) | 48.57 (±14.39) (n=58) | 57.22 (±9.40) | **T(85.37)=-3.44; p=0.001** |
| CDSS | 4.32 (±4.16) | 4.19 (±4.15) | Z(924.00)=-0.17; p=0.867 |
| Number of participants with OCS (Y-BOCS ≥ 8) | 28 (47.5%) | 8 (25.0%) | **x^2^(1)=4.38; p=0.036** |
| Number of participants with OCD (Y-BOCS ≥ 13) | 20 (33.9%) | 5 (15.6%) | x^2^(1)=3.48; p=0.062 |
| Inpatient Setting (n=59) | 17 (28.8%) | no data available |  |

Abbreviations

- CDSS = Calgary Depression Scale for Schizophrenia
- CGI = Clinical Global Impression
- CLZ = Clozapine
- GAF = Global Assessment of Functioning-Scale
- OCD = Obsessive-compulsive Disorder
- OCS = Obsessive-compulsive Symptoms
- PANSS = Positive and negative Symptoms Scale
- Psychosis NOS = Psychosis Not otherwise specified
- Y-BOCS = Yale-Brown Obsessive-Compulsive Scale


**STable 2 -** Correlations between phenotypic characteristics of participants

|  | **Y-BOCS Total Score** | **PANSS Total Score** | **PANSS Negative Items** | **PANSS Positive Items** | **PANSS General Items** | **GAF** | **Duration of CLZ in years** | **Prescribed dosage CLZ** |
| --- | --- | --- | --- | --- | --- | --- | --- | --- |
| **Y-BOCS Total Score** | n=91 | r=0.17; p=0.100 | r=-0.03; p=0.789 | r=0.16; p=0.122 | **r=0.23; p=0.028** | r=-0.18; p=0.085 | **r=0.28; p=0.008** | r=-0.03; p=0.815 |
| **PANSS Total Score** | r=0.17; p=0.100 | n=91 | **r=0.74; p<0.001** | **r=0.84; p<0.001** | **r=0.92; p<0.001** | **r=-0.77; p<0.001** | r=0.03; p=0.791 | r=0.20; p=0.054 |
| **PANSS Negative Items** | r=-0.03; p=0.789 | **r=0.74; p<0.001** | n=91 | **r=0.45; p<0.001** | **r=0.52; p<0.001** | **r=-0.56; p<0.001** | r=-0.04; p=0.735 | r=0.17; p=0.119 |
| **PANSS Positive items** | r=0.16; p=0.122 | r=0.84; p<0.001 | r=0.45; p<0.001 | n=91 | **r=0.70; p<0.001** | **r=-0.74; p<0.001** | r=-0.05; p=0.632 | **r=0.21; p=0.046** |
| **PANSS General Items** | **r=0.23; p=0.028** | **r=0.92; p<0.001** | **r=0.52; p<0.001** | **r=0.70; p<0.001** | n=91 | **r=-0.68; p<0.001** | r=0.10; p=0.361 | r=0.16; p=0.122 |
| **CGI** | r=0.50; p=0.637 | **r=0.66; p<0.001** | **r=0.56; p<0.001** | **r=0.63; p<0.001** | **r=0.55; p<0.001** | **r=-0.82; p<0.001** | r=-0.10; p=0.372 | r=0.17; p=0.101 |
| **GAF** | r=-0.18; p=0.085 | **r=-0.77; p<0.001** | **r=-0.56; p<0.001** | **r=-0.74; p<0.001** | **r=-0.68; p<0.001** | n=90 | r=0.17; p=0.101 | r=-0.18; p=0.098 |
| **CDSS** | r=0.01; p=0.958 | **r=0.38; p<0.001** | **r=0.22; p=0.036** | r=0.20; p=0.052 | **r=0.45; p<0.001** | r=-0.23; p=0.031 | r=-0.10; p=0.366 | r=-0.08; p=0.436 |
| **Duration of CLZ in years** | **r=0.28; p=0.008** | r=0.03; p=0.791 | r=-0.04; p=0.735 | r=-0.05; p=0.632 | r=0.10; p=0.361 | r=0.17; p=0.101 | n=90 | r=-0.01; p=0.953 |
| **Prescribed dosage CLZ** | =-0.03; p=0.815 | r=0.20; p=0.054 | r=0.17; p=0.119 | **r=0.21; p=0.046** | r=0.16; p=0.122 | r=-0.18; p=0.098 | r=-0.01; p=0.953 | n=90 |

Abbreviations

- CDSS = Calgary Depression Scale for Schizophrenia
- CGI = Clinical Global Impression
- CLZ = Clozapine
- GAF = Global Assessment of Functioning-Scale
- OCD = Obsessive-compulsive Disorder
- OCS = Obsessive-compulsive Symptoms
- PANSS = Positive and negative Symptoms Scale
- Psychosis NOS = Psychosis Not otherwise specified
- Y-BOCS = Yale-Brown Obsessive-Compulsive Scale


1. Milnacipran. Escitalopram. Venlafaxine. Sertraline. Doxepin. Citalopram. Fluvoxamine. Paroxetin. Duloxetine. Amitriptyline [↑](#footnote-ref-1)
2. Valproate. Pregabalin. Levetiracetam. Lamotrigine. Lithium [↑](#footnote-ref-2)
3. Haloperidol. Pipamperone. Chlorprothixene. Promethazine. Melperon. Perazin. Levomepromazine. Ziprasidone. Loxapin. Flupentixol. [↑](#footnote-ref-3)
4. Paliperidone. Risperidone. Amisulprid. Aripiprazole. Olanzapine. Quetiapine. [↑](#footnote-ref-4)
5. Diazepam. Lorazepam. Zopiclone. Clobazam [↑](#footnote-ref-5)
